# Bioinformatics-based screening and validation of PANoptosis-related biomarkers in periodontitis

**DOI:** 10.3389/fcell.2025.1619002

**Published:** 2025-06-19

**Authors:** Qing Sun, JinYue Hu, RuYue Wang, ShuiXiang Guo, GeGe Zhang, Ao Lu, Xue Yang, LiNa Wang

**Affiliations:** ^1^ Department of Endodontics and Periodontics, School of Stomatology, Dalian Medical University, Dalian, Liaoning, China; ^2^ The Affiliated Stomatological Hospital of Dalian Medical University, School of Stomatology, Dalian, Liaoning, China; ^3^ Academician Laboratory of Immune and Oral Development & Regeneration, Dalian Medical University, Dalian, Liaoning, China

**Keywords:** bioinformatics, biomarkers, periodontitis, PANoptosis, ZBP1

## Abstract

**Background:**

Periodontitis is the most prevalent chronic inflammatory disease affecting the periodontal tissues. PANoptosis, a recently characterized form of programmed cell death, has been implicated in various pathological processes; however, its mechanistic role in periodontitis remains unclear. This study integrates multi-omics data and machine learning approaches to systematically identify and validate key PANoptosis-related biomarkers in periodontitis.

**Methods:**

Periodontitis-related microarray datasets (GSE16134 and GSE10334) were obtained from the GEO database, and PANoptosis-related genes were retrieved from GeneCards. Differential gene expression analysis was performed using the GSE16134 dataset, followed by weighted gene co-expression network analysis (WGCNA) to identify relevant gene modules. The intersection of differentially expressed genes and WGCNA modules was used to define differentially expressed PANoptosis-related genes (PRGs). Protein-protein interaction (PPI) networks of these PRGs were constructed using the STRING database and visualized with Cytoscape. Subnetworks were identified using the MCODE plugin. Key genes were selected based on integration with rank-sum test results. Functional enrichment analysis was performed for these key genes. Machine learning algorithms were then applied to screen for potential biomarkers. Diagnostic performance was assessed using receiver operating characteristic (ROC) curves and box plots. The relationship between selected biomarkers and immune cell infiltration was explored using the CIBERSORT algorithm. Finally, RT-qPCR was conducted to validate biomarker expression in clinical gingival tissue samples.

**Results:**

Through comprehensive bioinformatics analysis and literature review, ZBP1 was identified as a PANoptosis-related biomarker in periodontitis. RT-qPCR validation demonstrated that ZBP1 expression was significantly elevated in periodontitis tissues compared to healthy periodontal tissues (P < 0.05).

**Conclusion:**

This study provides bioinformatic evidence linking PANoptosis to periodontitis. ZBP1 was identified as a key PANoptosis-related biomarker, suggesting that periodontitis may involve activation of the ZBP1-mediated PANoptosome complex.

## 1 Introduction

Periodontitis is a chronic inflammatory disorder of the periodontal tissues, primarily initiated by a dysbiotic dental plaque biofilm ecosystem (Kwon et al., 2021). Clinically, it is characterized by gingival inflammation, clinical attachment loss, and irreversible alveolar bone resorption (Sanz et al., 2012). Without timely and appropriate therapeutic intervention, advanced stages of the disease often lead to tooth hypermobility and eventual exfoliation (Silva et al., 2019), resulting in significant deterioration of masticatory function and oral health-related quality of life. Epidemiological studies indicate that the global prevalence of periodontitis approached nearly 60% during the past decade (2011–2020), establishing it as one of the most common chronic diseases worldwide (Trindade et al., 2023). Moreover, periodontitis is closely associated with various systemic conditions, including adverse pregnancy outcomes, cardiovascular and respiratory diseases, Alzheimer’s disease, and certain cancers (Teles et al., 2022). These associations underscore the importance of early diagnosis and intervention in managing periodontitis.

PANoptosis is a recently characterized form of inflammatory programmed cell death governed by the PANoptosome complex, which integrates molecular features of pyroptosis, apoptosis, and necroptosis. Notably, PANoptosis cannot be attributed to any single classical cell death pathway, highlighting the intricate crosstalk between these mechanisms (Christgen et al., 2020). The PANoptosome is composed of three key components: sensors, adaptors, and effectors (Samir et al., 2020). To date, four distinct PANoptosome complexes have been identified: the Z-DNA binding protein 1 PANoptosome (ZBP1-PANoptosome), the absent in melanoma 2 PANoptosome (AIM2-PANoptosome), the receptor-interacting protein kinase 1 PANoptosome (RIPK1-PANoptosome), and the Nod-like receptor family pyrin domain containing 12 PANoptosome (NLRP12-PANoptosome) (Qi et al., 2023). PANoptosis has been implicated in a wide range of pathological conditions, including infectious diseases, neurological disorders, autoimmune diseases, and various cancers.

Recent studies have highlighted the dual role of PANoptosis in disease progression. On the one hand, it plays a protective role in anti-tumor immunity. For example, nuclear export inhibitors such as KPT-8602 can induce PANoptosis in tumor cells by retaining ADAR1-p150 in the nucleus, thereby alleviating its suppression of ZBP1-mediated cell death signaling (Camilli et al., 2023). Additionally, *Fusobacterium* nucleatum outer membrane vesicles (Fn-OMVs) combined with oncolytic herpes simplex virus (oHSV) have been shown to activate ZBP1-dependent PANoptosis, offering promising therapeutic strategies for cancer treatment (Wang et al., 2024). On the other hand, dysregulated PANoptosis can exacerbate inflammation. For instance, in hemolytic conditions such as malaria and sickle cell disease, heme accumulation activates the NLRP12-PANoptosome complex, leading to acute renal tubular necrosis (Navuluri et al., 2025). In allergic diseases like allergic bronchopulmonary aspergillosis (ABPA), fungal proteases stimulate the ZBP1-TAK1 axis to drive PANoptosis, suggesting that necroptosis inhibitors may serve as effective therapeutic agents (Smallwood et al., 2024). These findings underscore the context-dependent duality of PANoptosis, which can mediate both protective and pathogenic outcomes.

Emerging evidence also suggests a potential role for PANoptosis in periodontitis. Lipopolysaccharides (LPS) derived from *Porphyromonas gingivalis*, a keystone periodontal pathogen, have been shown to significantly upregulate core regulatory proteins of apoptotic, pyroptotic, and necroptotic pathways in various cell types, including macrophages, fibroblasts, and stem cells (Zhang et al., 2024). Additional studies have identified the presence of all three forms of programmed cell death in rodent models of periodontitis, with elevated levels of associated key proteins observed in human periodontitis tissues and gingival crevicular fluid (Jiang et al., 2021). Collectively, these experimental and clinical findings implicate PANoptosis as a potential pathogenic mechanism in periodontitis; however, the specific composition of the PANoptosome involved remains to be elucidated.

Bioinformatics, an interdisciplinary field combining mathematics, computer science, and biology, enables the extraction of meaningful biological insights from large-scale datasets. It has been extensively applied in biomedical research and diagnostics (van Kampen and Moerland, 2016). Currently, the clinical diagnosis of periodontitis largely depends on physical examination and radiographic evaluation, which are limited by their reliance on visible disease manifestations and often result in delayed detection at advanced stages. With continued advances in genomics and bioinformatics, several molecular biomarkers have been proposed for the early diagnosis of periodontitis, offering new avenues for improved clinical outcomes. However, the utility of PANoptosis-related biomarkers in this context remains underexplored and requires further investigation.

Therefore, this study aims to identify PANoptosis-related biomarkers involved in periodontitis using integrated bioinformatics approaches, with the goal of providing novel insights for early diagnosis, prevention, and therapeutic intervention in this prevalent inflammatory disease.

## 2 Methods

### 2.1 Data acquisition

Periodontitis-related microarray datasets were obtained from the Gene Expression Omnibus (GEO) database (https://www.ncbi.nlm.nih.gov/geo/). The training dataset, GSE16134, comprises 241 periodontitis samples and 69 healthy control samples, while the validation dataset, GSE10334, includes 183 periodontitis samples and 64 healthy control samples. PANoptosis-related genes (PRGs) were retrieved from the GeneCards database (https://www.genecards.org), and only genes labeled as “Protein Coding” were selected, yielding a total of 23 PRGs for subsequent analysis (Table 1).

**TABLE 1:** PANoptosis related genes.

Gene	References
ZBP1	Karki and Kanneganti (2023)
STING1	Cai et al. (2023)
AIM2	Lee et al. (2021)
SAMHD1	Cai et al. (2023)
CASP6	Zheng et al. (2020)
PYCARD	Shi et al. (2024)
NLRP3	Oh et al. (2023)
DNM1L	Zheng et al. (2024)
NINJ1	Han et al. (2024)
MAPK1	Zhou et al. (2022)
TNF	Hao et al. (2024)
CASP8	Li et al. (2024)
NFS1	Lin et al. (2022)
RIPK3	Samir et al. (2020)
MAPK3	Zhou et al. (2022)
CASP1	Christgen et al. (2020)
XIAP	Qi et al. (2024)
CDK1	Ren et al. (2022)
MEFV	Moghaddas et al. (2017)
FADD	Lee et al. (2021)
IFNG	Malireddi et al. (2021)
RIPK1	Bynigeri et al. (2024)
TUG1	Yao et al. (2022)

### 2.2 Differential gene expression analysis

Differential gene expression analysis between periodontitis and healthy control samples in the training dataset (GSE16134) was conducted using the “limma” package in R software (version 4.3.1). Genes with a P-value <0.05 and |log2 fold change (log2FC)| > 0.5 were considered differentially expressed genes (DEGs). The results were visualized using volcano plots and heatmaps generated with the “ggplot2” and “pheatmap” packages in R, respectively.

### 2.3 Gene set variation analysis

Gene Set Variation Analysis (GSVA) is a non-parametric, unsupervised statistical approach used to evaluate the enrichment of predefined gene sets in transcriptomic datasets (Hänzelmann et al., 2013). In this study, the “ssGSEA” function from the “GSVA” package in R was utilized to transform the gene expression matrix of the GSE16134 dataset into GSVA enrichment scores for PRGs. The Wilcoxon rank-sum test was applied to assess differences in GSVA scores between periodontitis and healthy control groups, thereby identifying significantly differentially expressed PRGs.

### 2.4 Weighted gene co-expression network analysis

Weighted Gene Co-Expression Network Analysis (WGCNA) was used to identify modules of highly correlated genes. This method clusters genes into modules based on expression similarity, helping to detect potential biomarkers or therapeutic targets (Langfelder and Horvath, 2008). In this study, the “WGCNA” package in R was used to construct a weighted gene co-expression network from the GSE16134 dataset. The module with the highest correlation coefficient was intersected with the DEGs to obtain the differentially expressed WGCNA-PRGs relevant to periodontitis.

### 2.5 Construction of the protein–protein interaction network

A protein–protein interaction (PPI) network of the differentially expressed WGCNA-PRGs was constructed using the STRING database (https://string-db.org). The resulting interaction network was visualized using Cytoscape software (version 3.10.0). To identify densely connected subnetworks, clustering analysis was performed using the Molecular Complex Detection (MCODE) plugin within Cytoscape.

### 2.6 Identification of key genes and functional enrichment analysis

The differentially expressed PRGs with significant results from the rank-sum test were merged with the highest-scoring genes from the MCODE clustering analysis to identify key genes related to periodontitis and PANoptosis.

To further investigate the biological functions and signaling pathways involving these genes, Gene Ontology (GO) and Kyoto Encyclopedia of Genes and Genomes (KEGG) enrichment analyses were performed usingthe “clusterProfiler” package in R. The GO enrichment analysis was used to study the enrichment of key genes in biological processes (BP), cellular components (CC), and molecular functions (MF), while the KEGG analysis aimed to identify the relevant signaling pathways of these key genes (Gaudet et al., 2021; Kanehisa and Goto, 2000). Enrichment results were filtered using a significance threshold of P-value < 0.05.

### 2.7 Machine learning for screening potential biomarkers

Machine learning is an automated data analysis method widely used in clinical research (Deo, 2015). To further identify potential PANoptosis-related biomarkers in periodontitis, Least Absolute Shrinkage and Selection Operator (LASSO) regression and Support Vector Machine–Recursive Feature Elimination (SVM-RFE) analyses were performed using the “glmnet” and “e1071” packages in R with 10-fold cross-validation. Additionally, random forest analysis was conducted using the “randomForest” package. Candidate biomarkers were selected by intersecting the gene sets identified through these three machine learning methods.

### 2.8 Validation of diagnostic ability of potential biomarkers

To visualize the expression differences of the identified biomarkers between periodontitis and healthy control groups, box plots were generated using the “ggpubr” package in R, enabling a clear comparison of expression levels across both the training and validation datasets.

The diagnostic potential of the identified potential biomarkers was further evaluated by constructing receiver operating characteristic (ROC) curves using the “pROC” package in R. The area under the curve (AUC) was calculated to quantify diagnostic accuracy. Potential biomarkers with AUC values ≥0.8 in both the training and validation datasets were considered to have strong diagnostic value for distinguishing periodontitis from healthy controls.

### 2.9 Immune infiltration analysis

Based on the training and validation datasets, the CIBERSORT deconvolution algorithm was applied to investigate differences in immune cell infiltration between the periodontitis and control groups. Additionally, correlations between the identified biomarkers and various immune cell types were calculated in both datasets. The results were visualized using the “ggplot2” and “heatmap” packages in R.

### 2.10 Real-time quantitative polymerase chain reaction (RT-qPCR) validation of biomarker expression

Clinical gingival tissue samples were collected from patients at the Affiliated Stomatological Hospital of Dalian Medical University between October 2024 and December 2024. The control group comprised healthy gingival tissues from patients undergoing orthodontic treatment or third molar extraction, while the experimental group included tissues from patients with periodontitis undergoing periodontal surgery or tooth extraction. A total of twelve samples (six per group) were included in the study. Ethical approval was granted by the Institutional Review Board of the Affiliated Stomatological Hospital of Dalian Medical University (Approval No. 2024005). The inclusion and exclusion criteria were as follows (Heitz-Mayfield, 2024):

Inclusion Criteria: (1) age between 18 and 70 years; (2) presence of clinical attachment loss (CAL) on at least two non-adjacent teeth, or CAL ≥3 mm on the labial/buccal or palatal/lingual surfaces of at least two teeth; and (3) probing pocket depth ≥3 mm on two or more teeth.

Exclusion Criteria: (1) the presence of systemic diseases; (2) pregnancy or lactation; and (3) the use of immunosuppressive agents, antibiotics, or anti-inflammatory medications within the past 3 months.

Total RNA was extracted from gingival tissues using the TRIzol method. Complementary DNA (cDNA) was synthesized from mRNA using the Evo M-MLV reverse transcription kit. Quantitative real-time PCR (RT-qPCR) was conducted using the SYBR Green Pro Taq HS qPCR kit, with GAPDH serving as the internal control. Primer sequences are provided in Table 2. Gene expression was quantified using the 2^−ΔΔCt^ method. Statistical analysis and visualization were performed using GraphPad Prism version 10.

**TABLE 2:** Primer sequences for target genes.

Primer	Sequence (5'→3′)
GAPDH	Forward:TGCAACCGGGAAGGAAATGA
	Reverse:GCATCACCCGGAGGAGAAAT
ZBP1	Forward:GTCTCTCCGACTCCTTGCAG
	Reverse:TGTTCAAGGTGGCCTTCTCT

## 3 Results

### 3.1 Differential gene expression analysis

**FIGURE 1:**
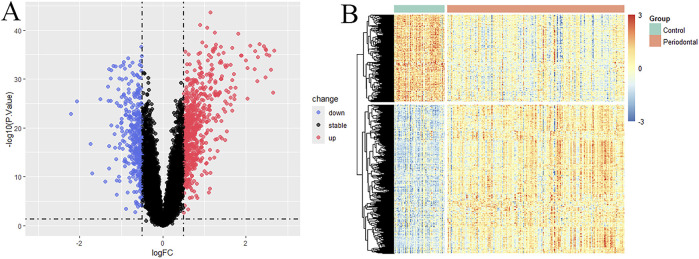
Differential gene analysis results. **(A)** Volcano plot of DEGs; **(B)** Heatmap of DEGs.

Differential gene expression analysis was conducted on the training dataset GSE16134, identifying a total of 1,067 differentially expressed genes (DEGs) between periodontitis and healthy samples. Among these, 673 genes were upregulated and 394 were downregulated, as illustrated in the volcano plot and heatmap (Figure 1).

### 3.2 GSVA analysis

**FIGURE 2:**
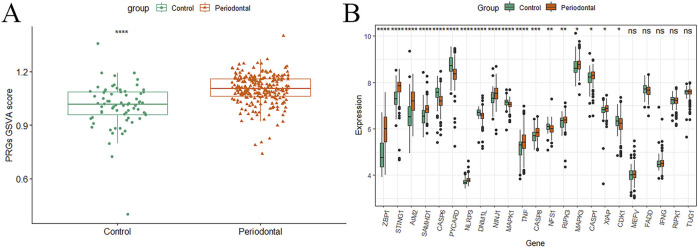
GSVA analysis results. **(A)** Boxplot of PRGs-GSVA score differences between the periodontitis group and the healthy control group; **(B)** Boxplot of rank sum test for differential expression of PRGs (*P < 0.05, **P < 0.01, ***P < 0.001, ****P < 0.0001).

The GSVA analysis revealed that the PRGs-GSVA scores were significantly higher in the periodontitis group compared to the healthy control group in the GSE16134 dataset, indicating that the biological process of PANoptosis is upregulated in periodontitis (P < 0.05; Figure 2A).

Furthermore, a rank-sum test identified significant differences for 18 genes—ZBP1, STING1, AIM2, SAMHD1, CASP6, PYCARD, NLRP3, DNM1L, NINJ1, MAPK1, TNF, CASP8, NFS1, RIPK3, MAPK3, CASP1, XAF1, and CDK1—between periodontitis and healthy control samples (*P* < 0.05; Figure 2B).

### 3.3 WGCNA analysis

**FIGURE 3:**
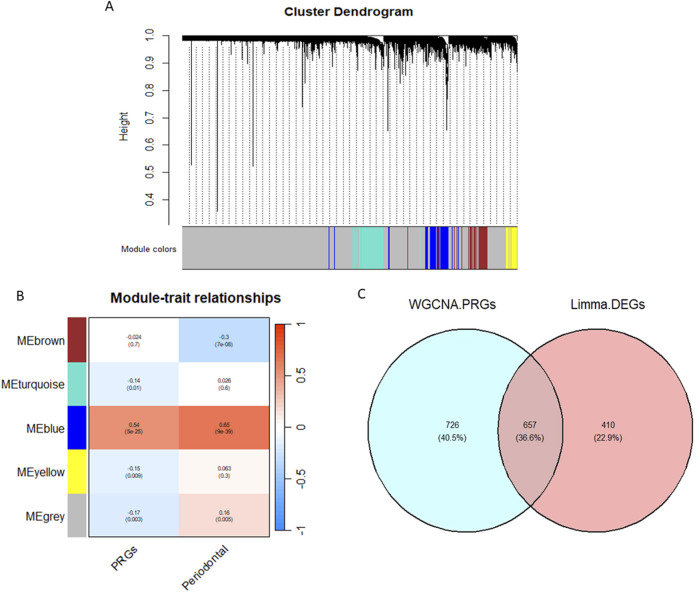
WGCNA analysis results. **(A)** Gene hierarchical clustering diagram; **(B)** Module correlation heatmap; **(C)** Venn diagram of WGCNA-PRGs and DEGs intersection.

WGCNA clustered genes with similar expression profiles into five modules using a cut height of 0.25. Among them, the MEblue module showed the strongest positive correlation with both periodontitis and PRGs. Genes from this module were considered key module genes. By intersecting them with the DEGs, 657 differentially expressed WGCNA-PRGs were identified for further analysis (Figure 3).

### 3.4 Construction of the PPI network

**FIGURE 4:**
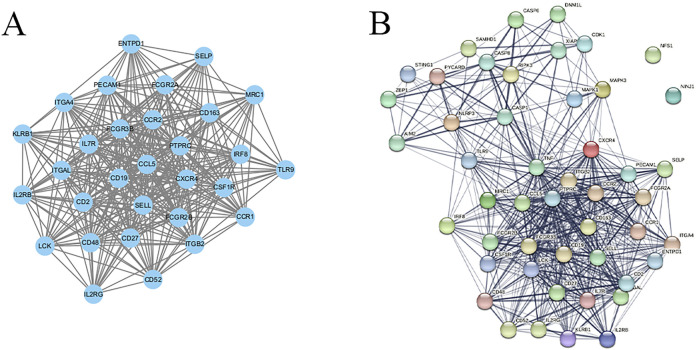
**(A)** PPI network of Cluster 1; **(B)** PPI network of 48 key genes.

The 657 identified WGCNA-PRGs were analyzed for PPI using the STRING database, and the results were visualized using Cytoscape. Clustering analysis via the MCODE plugin identified 18 interaction subnetworks, with Cluster 1 exhibiting the highest interaction score (24.138), comprising 30 genes: ITGB2, IL2RB, CSF1R, MRC1, CD19, ITGAL, CCL5, ITGA4, CXCR4, CD27, CD163, SELL, LCK, CCR1, IL7R, CCR2, FCGR3B, SELP, IL2RG, FCGR2B, KLRB1, CD2, PTPRC, CD48, ENTPD1, CD52, FCGR2A, TLR9, IRF8, and PECAM1 (Figure 4A).

These 30 genes were merged with the 18 PRGs that showed significant differential expression between groups, resulting in 48 PANoptosis-related genes in periodontitis. Their interactions were analyzed using the STRING database, revealing a network with 501 interaction pairs (Figure 4B).

### 3.5 Functional enrichment analysis of key genes

**FIGURE 5:**
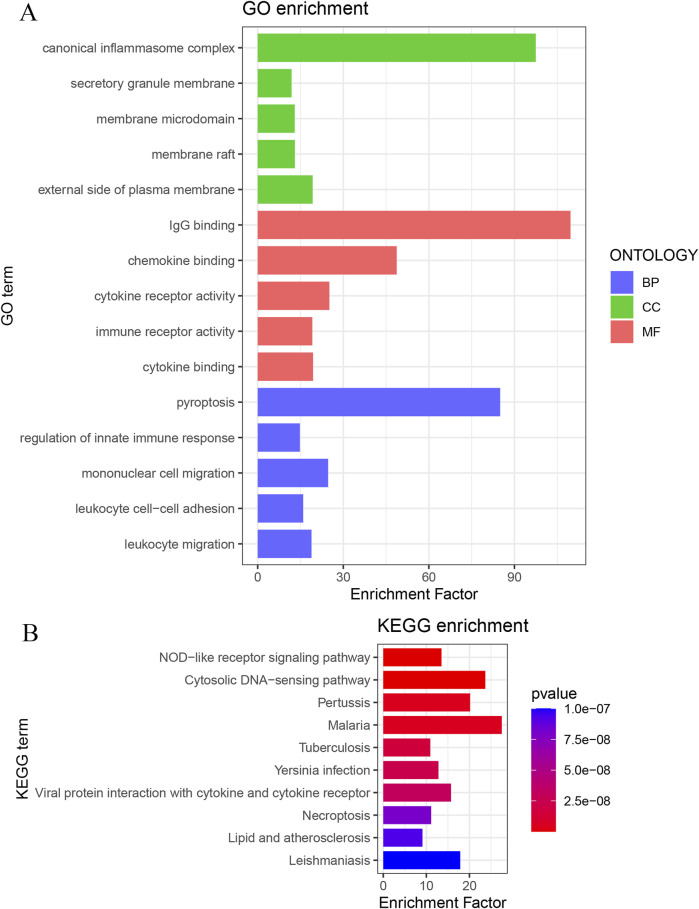
**(A)** GO enrichment analysis of key genes; **(B)** KEGG enrichment analysis of key genes.

Functional enrichment analysis of the 48 key genes was performed. GO analysis revealed 1,179 significantly enriched terms. In the BP category, 1,019 terms were enriched, focusing on pyroptosis, regulation of innate immune response, and mononuclear cell migration. In the CC category, 46 terms were enriched, including canonical inflammasome complex, external side of the plasma membrane, and secretory granule membrane. In the MF category, 114 terms were enriched, primarily related to IgG binding, chemokine binding, and cytokine receptor activity (Figure 5A). KEGG analysis identified 89 enriched pathways, such as the NOD-like receptor signaling pathway, cytosolic DNA-sensing pathway, and pertussis (Figure 5B).

### 3.6 Identification of potential biomarkers

**FIGURE 6:**
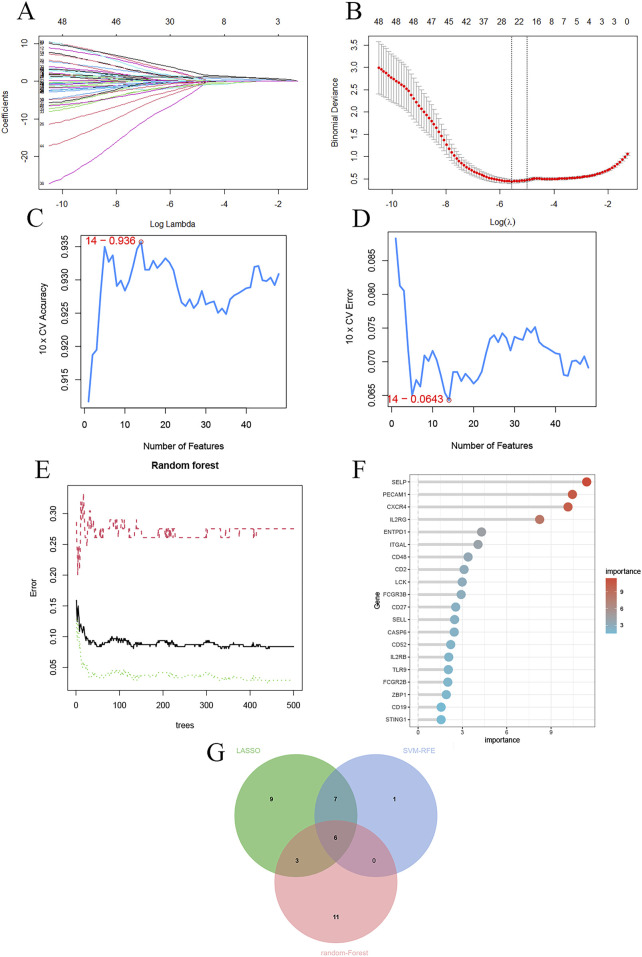
Machine learning identification of potential biomarkers. **(A)** LASSO regression coefficients; **(B)** Cross-validation curve; **(C)** SVM result accuracy; **(D)** SVM result error rate; **(E)** Random forest ntree value selection; **(F)** Variable importance ranking; **(G)** Venn diagram of the intersection of results from the three machine learning methods.

LASSO regression analysis was applied to the key genes, and the optimal lambda value yielded 25 biomarker candidates (Figures 6A,B).SVM-RFE analysis identified 14 feature genes with the lowest error and highest accuracy in 10-fold cross-validation (Figures 6C,D).Random forest analysis identified 20 feature genes with importance scores above 1.5 (Figures 6E,F).

The intersection of these three machine learning approaches revealed six potential biomarkers associated with PANoptosis in periodontitis: PECAM1, CXCR4, SELP, IL2RG, CD48, and ZBP1 (Figure 6G).

### 3.7 Validation of the diagnostic ability of potential biomarkers

**FIGURE 7:**
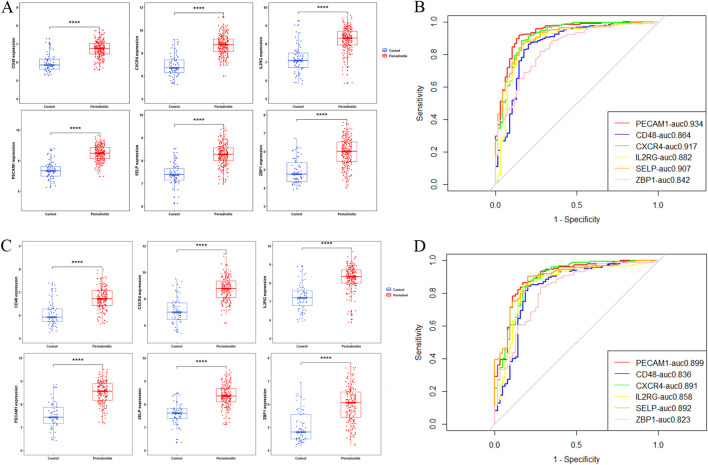
Differential expression analysis and ROC evaluation of candidate biomarkers. **(A,B)** Training set results; **(C,D)** Validation set results.

Box plot analysis confirmed that the expression levels of all six candidate biomarkers were significantly upregulated in periodontitis samples compared to healthy controls, in both the training and validation datasets (*P* < 0.05; Figures 7A,C).

ROC analysis showed that all six biomarkers achieved Area Under the Curve (AUC) values ≥0.8 in both datasets, indicating strong diagnostic performance (Figures 7B,D). Among these, SELP and PECAM1 were mainly expressed in endothelial cells and are involved in angiogenesis and cardiovascular disease (Liu et al., 2023; Privratsky et al., 2010). CXCR4 and IL2RG were predominantly expressed in lymphocytes, while CD48 was present in lymphocytes and other immune cells, contributing to immune regulation (Elishmereni and Levi-Schaffer, 2011; Le Floc’h et al., 2023; Nagashima et al., 2017). ZBP1 functions as an innate immune sensor critical for PANoptosis initiation. Therefore, ZBP1 was identified as a PANoptosis-related biomarker in periodontitis.

### 3.8 Immune cell infiltration analysis

**FIGURE 8:**
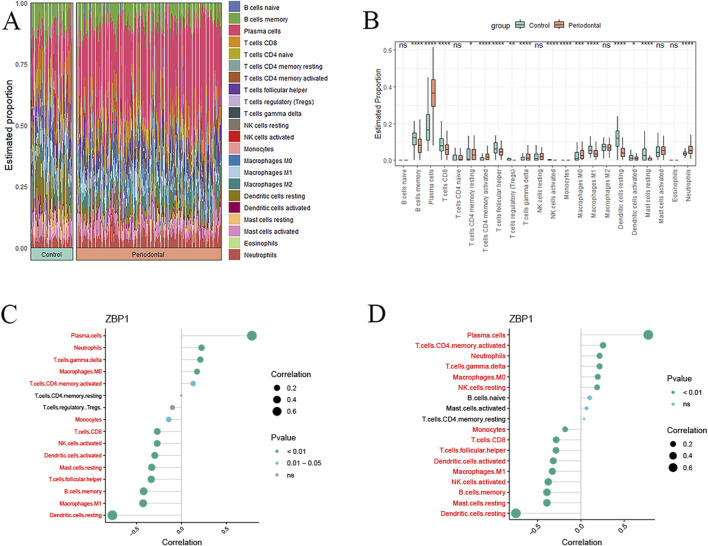
Immune infiltration analysis results. **(A)** Relative percentage of immune cell subpopulations in samples; **(B)** Differences in immune cell infiltration in samples (*P < 0.05, **P < 0.01, ***P < 0.001, ****P < 0.0001); **(C)** Biomarker-immune cell correlations (training set); **(D)** Biomarker-immune cell correlations (validation set).

CIBERSORT analysis revealed significantly higher infiltration levels of plasma cells, activated CD4^+^ memory T cells, γδ T cells, and neutrophils in the periodontitis group compared to healthy controls (*P* < 0.001; Figures 8A,B).

Correlation analysis demonstrated that ZBP1 was strongly positively correlated with plasma cells (r = 0.78), neutrophils (r = 0.22), γδ T cells (r = 0.21), and M0 macrophages (r = 0.17), while negatively associated with dendritic cells (r = −0.76) and M1 macrophages (r = −0.42) in the training cohort (P < 0.01; Figure 8C). These patterns were validated in the validation cohort, where ZBP1 again showed strong positive correlations with plasma cells (r = 0.77), γδ T cells (r = 0.21), neutrophils (r = 0.21), and M0 macrophages (r = 0.19), along with negative correlations with dendritic cells (r = −0.75) and M1 macrophages (r = −0.33) (*P < 0.01*; Figure 8D).

### 3.9 RT-qPCR validation

**FIGURE 9:**
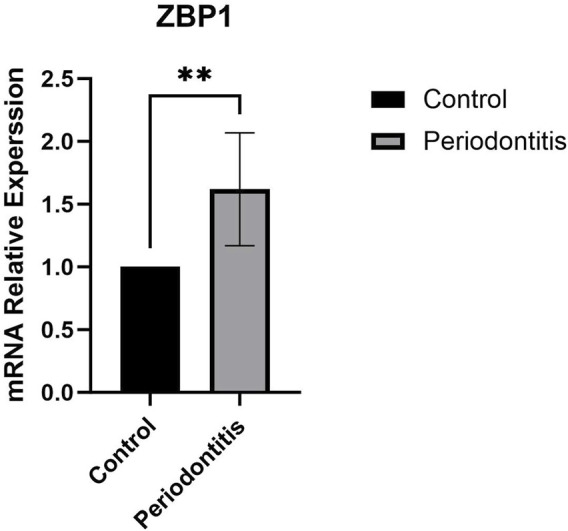
RT-qPCR results of ZBP1 (**P < 0.01).

RT-qPCR was performed on human clinical gingival tissue samples. The results showed that ZBP1 expression was significantly higher in periodontitis tissues than in healthy gingival tissues (*P* < 0.01; Figure 9).

## 4 Discussion

This study employed bioinformatics approaches to investigate the critical role of PANoptosis in periodontitis. GSVA analysis of the training dataset GSE16134 revealed upregulation of PRGs in periodontitis, indicating that PANoptosis is likely promoted in this condition. Differential gene expression analysis and WGCNA identified 48 key genes. GO enrichment analysis showed that, in terms of BP, these genes were associated with immune cell migration, adhesion, and regulation of innate immune responses. In the CC category, these genes were enriched in the external side of the plasma membrane, canonical inflammasome complexes, and secretory granule membranes. In terms of MF, they were primarily involved in IgG binding, chemokine binding, and cytokine receptor activity. KEGG pathway analysis indicated that these genes were enriched in the NOD-like receptor signaling pathway and the cytosolic DNA-sensing pathway. Collectively, these results suggest that the identified genes may contribute to the pathogenesis of periodontitis by activating inflammatory and immune-related pathways and by modulating the integrity and function of the cell membrane in periodontal tissues.

Using three machine learning algorithms, we identified six potential biomarkers. Literature review revealed that SELP and PECAM1 are associated with angiogenesis and cardiovascular diseases (Liu et al., 2023; Privratsky et al., 2010), while CXCR4, IL2RG, and CD48 are primarily involved in immune responses (Elishmereni and Levi-Schaffer, 2011; Le Floc’h et al., 2023; Nagashima et al., 2017). ZBP1, in particular, is closely related to PANoptosis and was thus selected as a potential biomarker in periodontitis. RT-qPCR validation using clinical gingival tissue samples confirmed that ZBP1 expression was significantly higher in periodontitis samples than in healthy controls (*P* < 0.01), suggesting its involvement in disease pathogenesis and highlighting its potential diagnostic value (AUC >0.80).

Z-DNA binding protein 1 (ZBP1), also known as DNA-dependent activator of interferon-regulatory factors (DAI) or DLM-1, is an innate immune sensor that plays a pivotal role in PANoptosis and antiviral immune responses. It was initially identified as a sensor of Influenza A Virus (IAV) infection (Kuriakose et al., 2016). ZBP1 contains two Z-DNA binding domains (Zα1 and Zα2), two receptor-interacting protein (RIP) homotypic interaction motifs (RHIM1 and RHIM2), and a C-terminal signaling domain (Zheng et al., 2020). The Zα2 domain of ZBP1 recruits key effectors such as RIPK3, RIPK1, CASP8, and CASP6 to assemble the ZBP1-PANoptosome. In addition, ZBP1 can form a ZBP1-NLRP3 inflammasome complex to mediate caspase-1 activation and IL-1β maturation, suggesting that inflammatory vesicle signalling and PANoptosis may co-exist in the pathological process of periodontitis (Oh and Lee, 2023; Zheng and Kanneganti, 2020). Recent studies have shown that oral pathogens such as *P. gingivalis* can activate ZBP1 in macrophages, leading to PANoptosis and exacerbated periodontal tissue destruction (Wu et al., 2025). These findings support the notion that microbial activation of ZBP1 may serve as a pathogenic driver of inflammatory cell death in periodontitis. *Fusobacterium nucleatum* has also been reported to activate ZBP1, triggering inflammation in murine models of apical periodontitis (Liu et al., 2022). Similarly, Sahingur et al. reported that the expression of DAI in human gingival tissue was 5.6-fold higher in chronic periodontitis samples than in healthy controls (Sahingur et al., 2013), consistent with our findings. Furthermore, rank-sum test analysis in this study revealed differential expression of key PANoptosome components such as RIPK3, CASP8, and CASP1, suggesting that periodontitis may involve ZBP1-mediated PANoptosome formation. However, further experimental validation is needed to clarify the underlying molecular mechanisms.

Periodontitis is a chronic inflammatory disease involving both innate and adaptive immune responses (Becerra-Ruiz et al., 2022). To further explore the immune microenvironment, CIBERSORT analysis was conducted. Compared to healthy controls, the periodontitis group exhibited significantly higher infiltration of plasma cells, activated CD4^+^ memory T cells, γδ T cells, and neutrophils, with plasma cells being the most prominent. Plasma cells, differentiated from B lymphocytes, are the dominant B cell type in periodontitis and regulate alveolar bone resorption via IL-35 and IL-37 production (Jing et al., 2019). Correlation analysis showed that ZBP1 was most strongly associated with plasma cells. Previous research has demonstrated that plasma cells express higher levels of ZBP1 mRNA than other B cell subsets. This co-expression pattern helps inhibit endogenous retrotransposons and viral infections, thus protecting the host from pathogen invasion (Herbert et al., 2022). Activated CD4^+^ memory T cells secrete pro-inflammatory cytokines such as IL-17 and IFN-γ, contributing to tissue destruction and inflammatory bone loss in periodontitis (Mahanonda et al., 2018). γδ T cells, a non-classical T cell subset, promote osteoclastogenesis and potentiate innate immune responses, thereby accelerating alveolar bone loss (Barel et al., 2022). Neutrophils, as key components of the innate immune system, are recruited and activated by periodontal pathogens. They release proteases and cytokines that intensify tissue damage and inflammation (Bassani et al., 2023). Furthermore, during Influenza A Virus (IAV) infection, viral replication activates ZBP1, which then triggers MLKL-mediated cell death. This form of regulated necrosis promotes neutrophil recruitment, further amplifying the inflammatory response (Zhang et al., 2020). The aforementioned studies indicate that both immune cells and biomarker contribute to the regulation of the immune microenvironment in periodontitis and play a crucial role in its pathophysiological processes.

Due to the good diagnostic ability of ZBP1 and its correlation with immune cells, ZBP1 may serve as a non-invasive biomarker detectable in salivary or crevicular fluid assays for the early screening and monitoring of periodontitis. Furthermore, as a core regulator of PANoptosis, ZBP1 represents a potential therapeutic target; small molecule inhibitors or pathway-specific modulators could be designed to suppress aberrant inflammatory cell death and immune cell recruitment in periodontitis.

Currently, research on ZBP1 as a diagnostic biomarker for periodontitis is limited. This study preliminarily demonstrates the diagnostic potential of ZBP1 in periodontitis and its possible involvement in PANoptosis; however, several limitations remain. First, the small clinical sample size and the absence of disease stage stratification may limit the generalizability of the findings. The dynamic expression profile of ZBP1 warrants further investigation through multicenter, large-scale studies incorporating clinical staging. Second, although bioinformatics analyses and RT-qPCR indicated co-expression of ZBP1 with PANoptosis-related genes, direct experimental evidence—such as immunoprecipitation or multiplex immunofluorescence staining—is lacking to confirm the formation of the ZBP1-PANoptosome complex in gingival tissues and to determine its precise localization. Finally, the clinical translational potential of ZBP1 remains underexplored. Further studies are needed to establish its utility as a definitive diagnostic and therapeutic target. Collectively, these limitations highlight the necessity of integrating mechanistic investigations, technological advancements, and clinical validation to comprehensively elucidate the functional network and translational prospects of ZBP1 in periodontitis.

## 5 Conclusion

Integrated bioinformatics analysis reveals that PANoptosis-associated genes potentially drive periodontitis progression through inflammatory and immune-related pathways. Notably, ZBP1 was identified as a PANoptosis-related biomarker, with disease pathogenesis potentially involving ZBP1-PANoptosome assembly. These findings position ZBP1 as a promising diagnostic biomarker and therapeutic target for periodontitis. However, large-scale experimental validation across expanded clinical cohorts is required to confirm these mechanistic insights.

## Data Availability

Publicly available datasets were analyzed in this study. This data can be found here: https://www.ncbi.nlm.nih.gov/geo/https://www.genecards.org.
